# High-Throughput 3D Phenotyping of Plant Shoot Apical Meristems From Tissue-Resolution Data

**DOI:** 10.3389/fpls.2022.827147

**Published:** 2022-04-18

**Authors:** Henrik Åhl, Yi Zhang, Henrik Jönsson

**Affiliations:** ^1^Sainsbury Laboratory, University of Cambridge, Cambridge, United Kingdom; ^2^Department of Applied Mathematics and Theoretical Physics, University of Cambridge, Cambridge, United Kingdom; ^3^Key Laboratory of Cell Proliferation and Regulation Biology of Ministry of Education, College of Life Science, Beijing Normal University, Beijing, China; ^4^Computational Biology and Biological Physics, Lund University, Lund, Sweden

**Keywords:** plant development, shoot apical meristem, flower development, 3D phenotyping, tissue segmentation, high-throughput, apex identification

## Abstract

Confocal imaging is a well-established method for investigating plant phenotypes on the tissue and organ level. However, many differences are difficult to assess by visual inspection and researchers rely extensively on *ad hoc* manual quantification techniques and qualitative assessment. Here we present a method for quantitatively phenotyping large samples of plant tissue morphologies using triangulated isosurfaces. We successfully demonstrate the applicability of the approach using confocal imaging of aerial organs in *Arabidopsis thaliana*. Automatic identification of flower primordia using the surface curvature as an indication of outgrowth allows for high-throughput quantification of divergence angles and further analysis of individual flowers. We demonstrate the throughput of our method by quantifying geometric features of 1065 flower primordia from 172 plants, comparing auxin transport mutants to wild type. Additionally, we find that a paraboloid provides a simple geometric parameterisation of the shoot inflorescence domain with few parameters. We utilise parameterisation methods to provide a computational comparison of the shoot apex defined by a fluorescent reporter of the central zone marker gene *CLAVATA3* with the apex defined by the paraboloid. Finally, we analyse the impact of mutations which alter mechanical properties on inflorescence dome curvature and compare the results with auxin transport mutants. Our results suggest that region-specific expression domains of genes regulating cell wall biosynthesis and local auxin transport can be important in maintaining the wildtype tissue shape. Altogether, our results indicate a general approach to parameterise and quantify plant development in 3D, which is applicable also in cases where data resolution is limited, and cell segmentation not possible. This enables researchers to address fundamental questions of plant development by quantitative phenotyping with high throughput, consistency and reproducibility.

## Introduction

Confocal imaging is a widely used tool in investigating spatiotemporal plant development at tissue, cell and subcellular resolution (Prunet et al., 2016). However, limitations in analysis tools and computational limitations have long restricted researchers from extensively analysing many aspects of development, particularly in three or more dimensions. This is especially pronounced for quantitative analysis of large datasets. Whilst some tools exist for segmenting tissues on the single-cell level (Fernandez et al., 2010; Berg et al., 2019; Wolny et al., 2020; Refahi et al., 2021), this approach requires data of particularly high quality and resolution, which is a limiting factor in terms of acquisition time, storage, and processing capabilities. In addition, a recurring issue in cell segmentation contexts is the usage of tissue dyes such as propidium iodide (PI) which stains entire cells upon tissue damage and cell death (Jones et al., 2016), rendering cell segmentation difficult or impossible. When analysing tissue-level properties, researchers thus often settle with acquiring data of intermediate to low resolution, on which manual analysis of tissue-level components can then be conducted (Lee et al., 2009; Bartrina et al., 2011; Li et al., 2012; Jones et al., 2016).

Whilst recent advancements have enabled new types of analyses on the cell level, few tools exist for complementing these data with information on which tissue-level substructures this information relates to. For situations where manual approaches can help, the issue of reproducibility and consistency becomes a limiting factor, and often analysis is restricted to 2D (Savaldi-Goldstein et al., 2007; Landrein et al., 2018). Frequently, analysis is limited to the tissue surface when working with 3D tissues. This approach makes computational aspects more efficient, allowing the measurement of geometrical features and the projection of fluorescent signals onto the surface mesh. However, available tools for surface extractions have limitations for curved surfaces (Barbier de Reuille et al., 2015), or do not provide a set of phenotyping methods for such surfaces (Kiss et al., 2017).

Animal studies have a successful record of using quantitative shape representation methods to enable more high-throughput analyses, particularly of cell and nuclear shapes (Martínez-Abadías et al., 2016; Ruan and Murphy, 2019; Medyukhina et al., 2020). However, such approaches have seen limited application in plant studies, particularly in 3D, despite the highly plastic and modular way in which plants develop.

Typically, the development of plant tissues involves repeated initiation of complex morphologies from typically more geometrically simplistic meristem domains. One such domain is the shoot apical meristem (SAM), which forms the basis for development of all aerial tissues, such as flowers. Cell growth and proliferation in the SAM is governed by a small pool of stem cells located in the central zone (CZ) at the very apex of the SAM, typically identified by the stem cell marker *CLAVATA3* (*CLV3*) (Fletcher et al., 1999). Several methods for identifying coordinates for the shoot apex using *CLV3* expression or geometrical means have been presented, but no standardised parameterisation method has been proposed (Willis et al., 2016; Galvan-Ampudia et al., 2020). In addition, studies have repeatedly approximated the SAM using quadric shapes (Itoh et al., 2000; Bozorg et al., 2014; Gruel et al., 2016), but the extent to which such an approximation captures the morphology of the SAM in 3D has not been thoroughly studied.

Flower development, which takes place at the periphery of the SAM, is also governed by modular principles. In the flower, the sequential formation of early flower buds, *primordia*, the initiation of the flower pedicel, and the primordial sepals are all part of initiations underpinning early organ development. As such, the initial stages of flower development are typically separated into the initiation of a primordial bud at the meristem periphery (Stage 1), the separation of the bud from the meristem and initiation of the pedicel (Stage 2), the initiation of sepal primordia on the flanks of the flower bud (Stage 3), and the separation and overgrowth of the sepals relative to the flower primordia (Stage 4) (Smyth et al., 1990).

Ultimately, the initiation of these complex morphologies depends on a multitude of genetic, hormonal, and mechanical signals. In terms of molecular signalling, the accumulation of the phytohormone auxin in local maxima has long been characterised as a primary mechanism for organ initiation (Okada et al., 1991). Auxin is transported to these maxima largely by membrane-bound transport-mediating proteins from the *PIN-FORMED* (*PIN*) gene family (Kuhlemeier and Reinhardt, 2001; Heisler et al., 2005). Several PIN proteins polarise on the cell membrane, leading to directional cellular efflux of auxin. PIN1 is the primary auxin transporter in the SAM, where the polarisation pattern denotes the sites for incipient primordia (Kuhlemeier and Reinhardt, 2001; Heisler et al., 2005). Consequently, *pin1* defective mutants are unable to grow floral organs, and instead form naked, pin-like stems (Okada et al., 1991). Additionally, plants treated with Naphthylphthalamic acid (NPA), which associates with and inhibits PINs, form a similarly organless phenotype (Thomson et al., 1973; Abas et al., 2021).

In contrast to *PIN*s, *AUXIN RESISTANT 1 (AUX1)* and members of the *LIKE-AUX* (*LAX*) family are responsible for cellular influx of auxin. In particular, the quadruple *aux1lax1lax2lax3* mutant has been identified to have aberrant floral phyllotaxis (Bainbridge et al., 2008), whereas the singular *aux1* mutant has been described to have a leaf phyllotaxis defect (Stieger et al., 2002). Whilst PIN and AUX1/LAX proteins are important short-range focusers of auxin transport, the *ATP-binding cassette B* (*ABCB*) gene family transporters *ABCB1* and *19*, which are membrane-bound and apolar efflux transporters, have been described as long-range transporters of auxin. These have been implicated in processes such as shoot branching (Bennett et al., 2016).

Whilst *PIN1* is a known primary regulator of phyllotaxis, mutations in other *PIN*s have not been described to impact phyllotaxis or shoot morphology in the inflorescence. Similarly, whether perturbations of *ABCB1* and *ABCB19* affect floral phyllotaxis as well as shoot and flower morphology is unknown. In addition, whereas knockouts of the AUX1/LAX influx proteins are known to affect the robustness in phyllotaxis, little is known about the impact of shoot morphology as a whole, as well as the extent of these properties at the very earliest stages of organ initiation.

In terms of mechanical regulation of development, the shoot epidermis has repeatedly been implicated as a mechanical regulator of shape development (Savaldi-Goldstein et al., 2007; Hamant et al., 2008), where tissue-wide stress and strain patterning, alongside local growth help to guide the formation of the different developmental stages (Hamant et al., 2008; Refahi et al., 2021). Cell and tissue mechanics are governed by interactions between the cell wall, plasma membrane, and cytoskeleton. As such, cytoskeleton and cell wall biosynthesis are integral components for regulating growth and maintaining cell and tissue integrity (Landrein and Hamant, 2013). Recent studies have identified that certain mechanical regulators appear in different types of expression domains in the SAM (Yang et al., 2016); for example, members of the *CELLULOSE SYNTHASE* (*CesA*) gene family, which are vital regulators of cellulose biosynthesis, are commonly either uniformly expressed in the outer cell layers of the shoot (Type 1), or primordia-specific (Type 2). In particular *CesA1* and *3* have Type 1 expression patterns in the SAM, whereas *CesA6* is of Type 2 (Yang et al., 2016). Similarly, the Xyloglucan 6-xylosyltransferase encoding family (*XXT*) can be expressed either as Type 1 (*XXT1* and *5*), or Type 2 (*XXT2*) (Yang et al., 2016). However, the impact of these differential expression domains in regulating shape and morphogenesis on the tissue level has not been thoroughly studied.

Here, we introduce a framework for generating 2.5D epidermal tissue surfaces from binary masks generated from confocal data, which is flexible with respect to the tissue orientation defined by the acquisition angle. Further, we utilise the vertex-level curvature of this tissue together with a gradient descent method to identify and label substructures in the tissue. Our method is applicable on data of varying resolution and quality, and thus enables the analysis of tissue samples also in cases where the tissue has been damaged, or when the resolution is insufficient to conduct cell-level segmentation and analysis.

We illustrate the potential of our method by showcasing examples including both geometric and genetic data, and show how a mathematical paraboloid can be mapped to the inflorescence domain of the SAM in order to quantify tissue-wide curvatures with few parameters, and to identify the geometric apex of the inflorescence shoot. Further, we apply our method to illustrate differences in the positioning and other tissue-level geometric properties of initiating flower organs, as well as the inflorescence domains in two different datasets.

## Results

### A Robust and Modular Pipeline for Surface Generation of Shoot Apical Meristems and Other Plant Tissues

**FIGURE 1:**
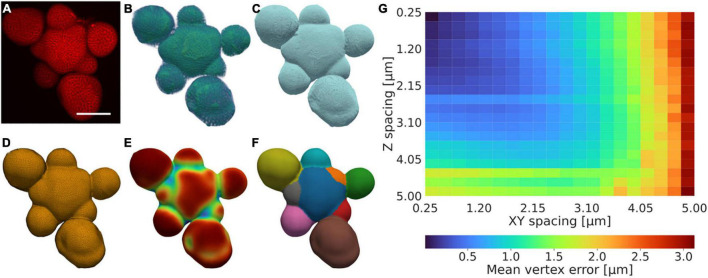
A low-resolution tolerant surface quantification framework. **(A–F)** Example illustration of the quantification framework, showing the fundamental stages illustrated by the **(A)** raw confocal intensity data after acquisition, **(B)** intensity data after pre-processing, **(C)** contour after post-processing, **(D)** mesh after post-processing, **(E)** vertex curvature after post-processing, and **(F)** segmentation after post-processing. **(A)** is a summed intensity projection, **(B,C)** are 3D volume renders of the data, **(D–F)** are 2.5D surface meshes encapsulated in 3D space. Scale bar: 75 μm. Scales in **(B–F)** further to **(A)**. Colouring in **(A,B)** and **(E)** shows the corresponding value magnitude in arbitrary units, whereas **(C,D)** are false-coloured, and **(F)** is coloured by integer label value. A detailed description of the protocol is provided in Methods and further illustrated in Supplementary Figure 1. **(G)** Mean error for the closest vertex on the downscaled mesh for every vertex in the high-resolution mesh. The error increases more rapidly with decreasing resolution in XY relative to that of Z as the X and Y dimensions are altered synchronously.

Our protocol for generating triangulated isosurfaces from 3D confocal data relies on a series of acquisition, preprocessing, contouring, meshing, and mesh post-processing steps, optionally followed by segmentation and segmentation post-processing (Figures 1A–F and Supplementary Figure 1, Methods). The pipeline utilises a morphological snake step to generate an initial contour, which is intensity-based, orientation agnostic, robust to noise, and does not require that object boundaries are well-defined (Chan and Vese, 2001; Márquez-Neila et al., 2014). This method is extended by adding additional heuristical and case-specific methods relating to the specific data type and tissue orientation to aid the quality of the quantification, both in terms of the contouring and surface generation steps (Methods). This allows for the quantification of tissues and the utilisation of data also when is limited; for example, autofluorescence or damaged tissues can be used provided the corresponding signal is sufficiently representative of the tissue morphology (Supplementary Figure 2). To illustrate the versatility of our method, realistic and accurate surface meshes of a set of five different *Arabidopsis thaliana* tissues with varying morphologies were generated (Figures 1A–F and Supplementary Figure 3).

To analyse the sensitivity of the method relative to the data resolution, the method was applied to an example tissue with complex morphology, with increasing downsampling in the Z and joint XY dimensions, respectively. The method produces a surface estimation typically falling within an error of 0.5 μm also in cases when the spatial resolution is relatively low (∼1 μm), and produces an average error consistently lower than the corresponding image resolution (Figure 1G).

### A Fitted Paraboloid Accurately Approximates the Local Wild Type Shoot Shape and Apex

Being able to parameterise a shape using a few variables can be important for an efficient high-throughput quantitative analysis and comparison of tissue shapes. Because of this, we sought to resolve whether a quadric shape is representative of the inflorescence shoot morphology, both for the local growth occurring in the CZ, and for the shoot and stem altogether. To that end, we implemented a method for quantifying 3D dome shape that revolves around the mapping of a mathematical paraboloid to the inflorescence domain (Methods). We applied our method to extract the inflorescence domain from *Arabidopsis thaliana* plants grown either on plates with media containing NPA (Willis et al., 2017), i.e., without forming flower primordia, or without NPA on soil (Methods).

To separate the inflorescence meristem domain from early flower tissue, we implemented a segmentation protocol applicable to our surface meshes (Figures 1A–F and Supplementary Figure 1). The mean curvature was calculated at all vertices in the mesh, and the curvature field then smoothened to amplify high (ridges) and low (trenches) curvature regions (Methods). Trenches typically correspond to boundary regions between different organs, such as the inflorescence meristem and emerging flower primordia, and ridges typically denote apex points of morphologically relevant organs, such as flower primordia. The curvature field was used to identify basins of attraction within the curvature values, i.e., points to which multiple trajectories end up when iteratively connecting vertices to their vertex neighbour with the highest curvature (Methods). Additional merging of neighbouring attractors were used to identify organs with correct classification (Methods).

**FIGURE 2:**
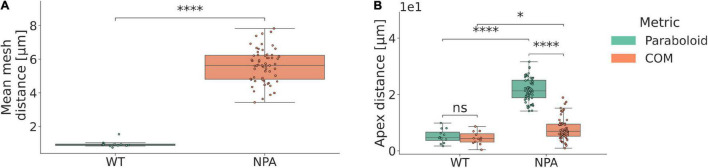
Local shoot meristem growth is well approximated by a paraboloid. **(A)** Mean euclidean mesh distance in terms of identification of the closest vertex on the paraboloid, for every point in the surface mesh. Paraboloids are good approximations of the inflorescence tissue shape in WT plants. μ_WT_ = 0.96 μm; μ_NPA_ = 5.62 μm; d = 4.66 μm; *p* = 5.84e-08 (****); n_WT_ = 12; n_NPA_ = 59; where μ represents the mean, d the corresponding difference between the means, and n the number of samples per category. **(B)** Euclidean distance between the identified apices, using either the fitted paraboloid (para), or the inflorescence centre of mass (COM). Paraboloid and centre of mass methods accurately predict the *CLV3* peak expression domain. μ_WT,para_ = 5.19 μm; μ_NPA,para_ = 21.69 μm; d_para_ = 16.50 μm; μ_WT, COM_ = 4.51 μm; μ_NPA,COM_ = 7.72 μm; d_COM_ = 3.21 μm; p_WT_ = 1.00 (ns); p_NPA_ = 2.65e-19 (****); p_para_ = 2.33e-07 (****); p_COM_ = 2.1e-02 (*). The centre of mass, paraboloid apex and *CLV3* coordinates are projected onto the mesh using the paraboloid central axis for comparison purposes. See Methods and Supplementary Figure 4 for further descriptions of the apex quantification method.

When extracting the inflorescence domains and mapping paraboloids to them (Supplementary Movie 1), it was observed that wild type (WT) inflorescence domains are significantly better approximated by paraboloids than organless NPA-treated shoots (Figure 2A). The average distance between the paraboloid and the actual surface is less than 1 μm in the WT plants (Figure 2A).

In order to assess whether the geometric apex defined by the mapped paraboloid corresponds to the central zone as defined by *CLV3* expression, we investigated plants expressing a *pCLV3::dsRed x myr-YFP* transgene. We then utilised the centre of mass (COM) of the voxels with the highest *CLV3* signal, projected onto the surface mesh, as representative of the central zone (Supplementary Figure 4, Methods). We found that the geometric apex, both as defined by the paraboloid and by using the inflorescence tissue COM, correlates well with the position of the *CLV3* expression in WT plants. The geometrical apex is predicted on average within a cell size from the centre of the central zone, as the average distance is 4.51 μm in WT plants (Figure 2B). In contrast, the paraboloid is not as accurate when applied to NPA treated shoots (Figure 2B). In NPA-grown plants, the tissue COM method is at large better than the paraboloid method (Figure 2B). This is indicating that the paraboloid apex typically aligns more with the tissue periphery, whereas the tissue COM-defined apex correlates better with the apex defined by the *CLV3* peak expression.

In summary, the paraboloid is a good approximation of the local growth domain and the geometric apex in WT plants, but does not capture the combined shape of the shoot apex and periphery well in the NPA-treated plants.

### The Inflorescence Dome Shape Is Controlled by Local Regulatory Expression Patterns

To further illustrate the applicability of our framework, we again utilised the *Arabidopsis thaliana* SAM as a case study in extracting tissue-level information for large-scale phenotyping. Specifically, we investigated the impact of hormonal and mechanical perturbations on inflorescence dome shape. Dome shape analysis was conducted on two datasets, which are referred to as the auxin transport and mechanical datasets. The auxin transport dataset was generated from the imaging of auxin transport mutants *abcb1*, *abcb19*, *abcb1,19*, *aux1*, and *aux1lax1,2,3* (Supplementary Table 1, Supplementary Figure 5, Methods), which are not canonically implicated as direct or primary regulators of phyllotaxis and shoot morphology. We abbreviate genotypes with multiple mutations within the same gene family using comma-notation (Supplementary Table 1). The mechanical dataset was obtained from mutants with perturbed cell wall biosynthesis pathways, and consists of *cesa1^any1^*, *cesa3^eli1^*, *cesa3^je5^*, *cesa6^prc1–1^*, *xxt1,2*, and *xxt1,2,5* (Supplementary Table 1, Methods). Using the surface generation pipeline, meshes were generated, and a paraboloid subsequently fitted. The tissue-level shoot curvature was then computed from the principal curvatures defining the paraboloid (Methods).

**FIGURE 3:**
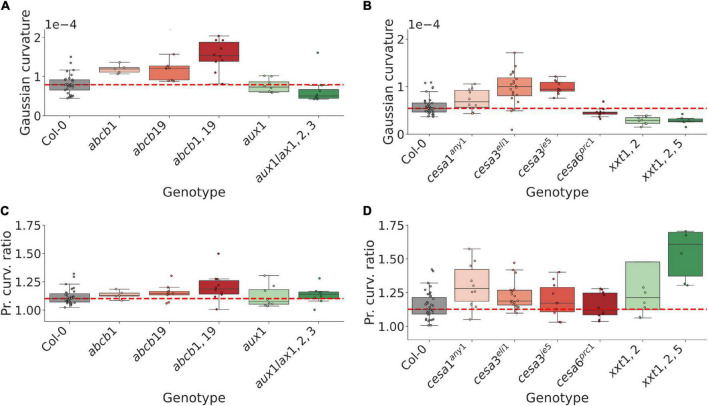
Mutations in gene homologs can result in opposite shoot curvature phenotypes, suggesting an important role in region-specific expression for maintaining shoot morphology. **(A)** Tissue-level gaussian curvature of the shoot inflorescence in WT and 5 auxin transport related mutant genotypes. Cumulative mutations in the *ABCB* efflux transporter family lead to more pointed shoots, whereas multiple mutations in the *AUX1/LAX* influx transporter family lead to flatter shoots. **(B)** Same as for **(A)**, but for WT and 6 mechanically perturbed genotypes. Mutations in different *CesA* genes can lead to opposite shoot phenotypes, suggesting a role in region-specific expression or regulation in maintaining the inflorescence shape. **(C)** Max/min ratio of the curvatures defining the corresponding fitted paraboloid (Methods), indicating curvature symmetry, for the auxin transport mutant dataset. At large, no strong trends are apparent, although cumulative mutations in the *ABCB* efflux transporters appear to trend toward less symmetric shoots with statistically significant differences (cf. Supplementary Figure 6E). **(D)** Same as in **(C)** for the mechanical mutant dataset. Perturbations of the mechanical mutants generally lead to less symmetric shoots. Significance tests are reported in Supplementary Figure 6.

Both the auxin transport and the mechanical datasets exhibit a mixture of shoot curvature phenotypes, ranging from negative to positive shifts in Gaussian curvature relative to WT plants (Figures 3A,B and Supplementary Figures 6C,D). Several mutants in the mechanical dataset exhibit a tendency to grow in less symmetric shapes compared to WT (Figure 3D and Supplementary Figure 6F), and in the range of mutants analysed in this study, the mutants in the mechanical dataset are generally more anisotropic than mutants in the auxin transport dataset (Figures 3C,D and Supplementary Figures 6E,F).

Whilst *ABCB1* and *19* have not been described as regulators of shoot morphology, both the shoot curvature and asymmetry measures trend toward increasing values when the genes are perturbed (Figures 3A,C and Supplementary Figures 6C,E). To better understand a possible connection to the shoot phenotype we imaged SAMs using a transgenic reporter (pABCB19::ABCB19-GFP). *ABCB19* is expressed in early flower primordia (Supplementary Figure 5A), suggesting a potential role in regulating shoot shape by adjusting tissue-wide auxin efflux from primordia during flower initiation. In contrast, multiple mutations in the established shoot-localised *AUX1/LAX* influx carriers, which are expressed predominantly in the CZ and provasculature (Heisler and Jönsson, 2006; Bainbridge et al., 2008), result in a flatter dome (Figure 3A and Supplementary Figure 6C). These results suggest that the shoot localised transport proteins and their expression patterns have a role in maintaining the inflorescence meristem shape by controlling auxin movement and patterning within the shoot and its peripheral organs.

Mutations in the *CesA3*, which is largely uniformly expressed across the shoot outermost cell layers, give rise to more pointed shoots; a similar, non-significant trend is observed for the uniformly expressed *CesA1* (Figure 3B and Supplementary Figure 6D). In contrast, perturbed *CesA6*, which is predominantly expressed in primordia (Yang et al., 2016), leads to a flatter dome (Figure 3B and Supplementary Figure 6D). The double-mutation *xxt1,2*, which from their WT expression domains represent both a uniform and primordia-specific perturbation (Yang et al., 2016), exhibits a flatter dome, whereas an additional mutation in the uniformly expressed *XXT5* gene leads to a similar dome curvature (Figure 3B and Supplementary Figure 6D). These results suggest relative rigidity between the CZ and organ initiation sites, as well as the overall tissue integrity in general, is important in controlling the resulting dome shape.

### High-Throughput Quantification and Extraction of Organ-Level Features Elucidate Developmental Dynamics of Early Flower Organ Development

**FIGURE 4:**
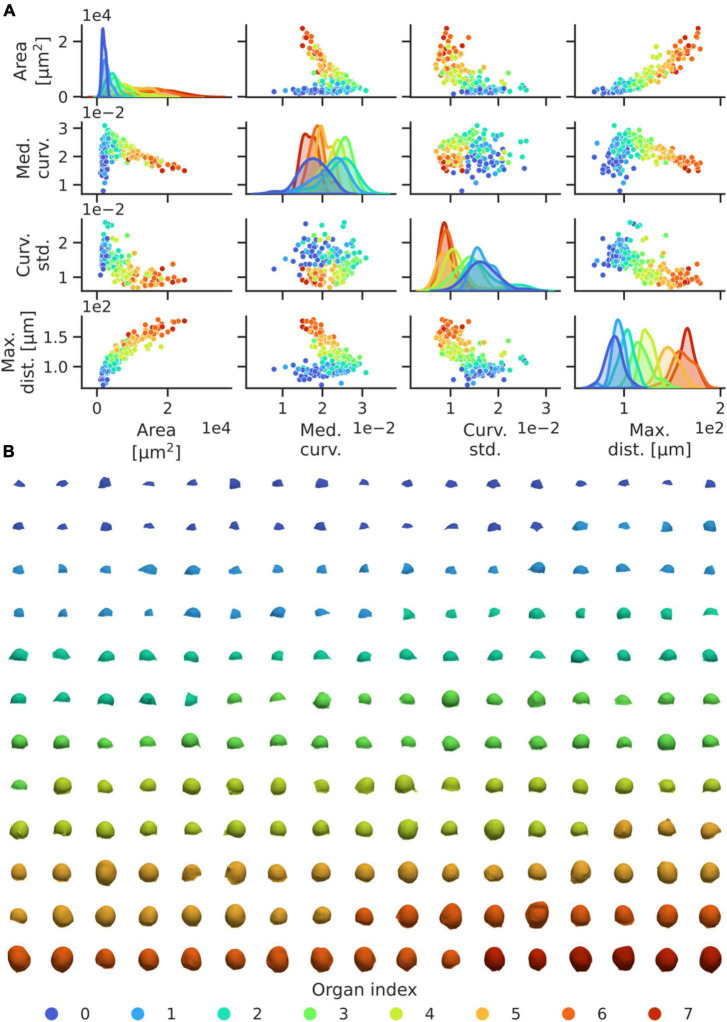
High-throughput geometric feature quantification of WT flower organs enable accurate identification of the development of, and relationship between, morphological features. **(A)** Pairwise dependencies on mesh surface area, median of the average vertex curvatures, standard deviation of the mean and maximal vertex distance relative to the inflorescence centre of mass for each organ in the dataset. To avoid outlier obfuscation, curvature statistics are truncated to be within the 10 and 90% distribution quantiles, per organ. Organ indices are coloured from the most to least recently initiated primordium. Kernel density estimates (on the diagonal) have areas normalised to 1 for each organ index, and the y-axis represents density. **(B)** Illustration of the segmented WT flower organs, ordered by left-right, top-bottom in coloured groups corresponding to their phyllotactic index. Generally, organs of a certain index exhibit similar morphology.

To identify patterns in the development of flower geometries, we utilised the segmented data from the 29 WT meristems included in the auxin transport dataset (Figure 3). Parameters of the pipeline were set such that the initial primordium identified roughly corresponds to the first identifiable organ of developmental stage 1 in WT plants, which is defined by early bulging of the tissue in the shoot periphery (Smyth et al., 1990). From the resulting segmentation, geometric data from 198 emerging flower primordia was extracted (Figure 4, Methods). The segmentation was thereafter complemented with manual identification of the phyllotactic order and corresponding spiral direction of the identified primordia. Through this, geometric features were related to the corresponding order of the organs relative to the first identified primordia per plant (Figure 4, Methods).

Flowers at any given order index are largely of similar morphology between different plants (Figure 4). In terms of the relationship between organ index and developmental stage, we note that organ indices 0–2 roughly correspond to stage 1 (early bulging), indices 2–4 to stage 2 (initiation of pedicel and separation from inflorescence), and 5–7 to stage 3 (substantial outgrowth and initiation of sepals) (Figure 4B; Smyth et al., 1990). Certain geometric measures, such as the domain surface area, the organ-wide median of the mean vertex curvature, the mean curvature standard deviation, and the maximal distance to the inflorescence centre of mass, are informative measures of organ development stages, and all measures exhibit clear trends in the development of these variables with the developmental stage of the organs (Figure 4A). Hence our shape quantification provides a high-resolution data-driven characterisation of early flower development.

A clear quantitative pattern in the transition between organ indices 2 and 3 can be identified, where the mean surface curvature rapidly decreases after an increase up until that stage (Figure 4A). Phenomenologically, this corresponds to the separation of primordia and inflorescence *via* the initiation of pedicel growth, i.e., the transition between development stages 1 and 2 (Figure 4B). This result is illustrative of the fact that our quantitative approach provides a method to extract 3D geometric data with high statistical accuracy, and to link quantitative data directly to morphological events. Whilst organ index categories overlap in terms of singular quantities, as expected given that there is no synchronisation in flower stages at the collection time and given the temporal variability in early flower development (Refahi et al., 2021), our results indicate the potential of our quantitative framework in refining traditional classifications of flower development stages and developmental dynamics within a reproducible analysis framework.

### Phyllotaxis Phenotyping of Auxin Transport Mutants Elucidates That Non-local Regulation Can Affect Phyllotaxis Robustness

To illustrate the potential applicability in phenotyping plant shoots with small phenotypic variability using our protocol, we used the segmented hormone dataset (Figure 3), and complemented the analysis with further perturbations in the auxin transporters *PIN3*, *4* and *7* which are not localised in the inflorescence shoot (Guenot et al., 2012). These genotypes consist of *pin3*, *pin4*, *pin7*, *pin3,4*, *pin3,7*, *pin4,7*, and *pin3,4,7* (Supplementary Table 1). In total, segmented isosurfaces from a total of 172 plants were successfully generated, for which the aforementioned organ ordering procedure was repeated. Following this, the corresponding divergence angles were calculated (Methods).

**FIGURE 5:**
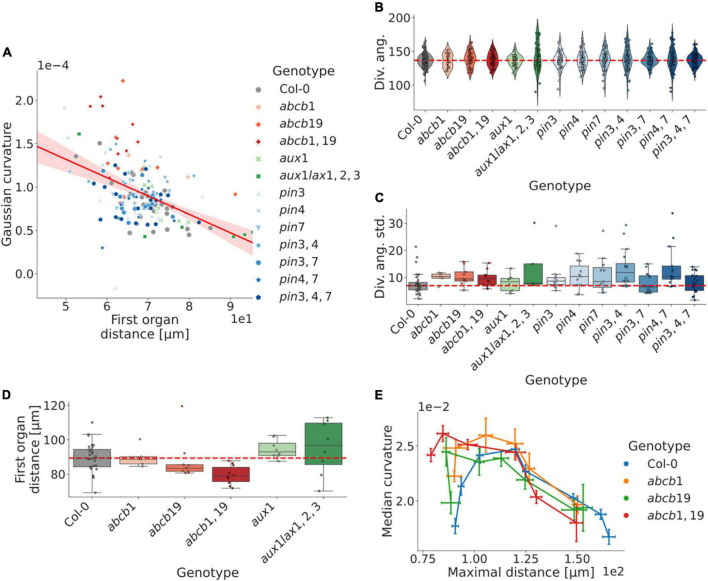
Robustness in phyllotaxis is dependent on non-local auxin regulation, whereas local auxin transport affects shoot morphology. **(A)** The inflorescence meristem curvature correlates with the first organ distance (least-squares regression: *r* = −0.48, *p* = 1.23e-10). Outliers further than 100 μm from the meristem apex removed from the analysis. **(B)** Distributions of divergence angles for WT and five auxin transport related mutant genotypes. Each data point represents the average divergence angle in a plant. Typically 4–8 organs can be identified per plant. Perturbed plants are more prone to include severely aberrant angles. **(C)** Per-plant distributions of standard deviations from the in divergence angles around the canonical WT average (137.5°). The majority of perturbed plants exhibit increased variability in the internal angles. **(D)** Size-distributions as defined by the distance from the inflorescence domain centre of mass to the first organ counterpart. Mutations in the *ABCB* efflux transporter genes trend toward smaller shoots. **(E)** Curvature-distance distributions for organs in the mutations in the *ABCB* efflux transporter genes, which affect the plastochron for earlier primordia. The maximal distance refers to the maximal euclidean distance for the organ vertices relative to the inflorescence apex. The corresponding organ index in perturbed plants is generally at a later developmental stage than its WT counterpart. Error bars in **(E)** show the standard error of the mean, whereas points represent the group means; lines are ordered in terms of the corresponding organ index. Dashed red lines represent the WT distribution median in **(B–D)**. Corresponding significance tests are reported in Supplementary Figure 6.

We first calculated the relationship between curvature and shoot size, using the apex to first organ distance as a proxy measure. Our analysis shows a strong negative correlation, with typically decreasing shoot curvature relative to the shoot size; however, the differing degrees of correlation between different genotypes indicate a distinct separation between the measures (Figure 5A). We observe that mutations in *ABCB1* and *19* exhibit an increasingly perturbed shoot morphology with cumulative mutations relative to WT (Figure 5D and Supplementary Figures 5B–E, 6B; cf. Figure 3A).

The divergence angles of the mutated genotypes exhibit similar distributions to WT plants, although mutated plants are more prone to have singular divergence angles that deviate severely from the WT mean (Figure 5B). In addition, the intraplant divergence angle standard deviation is higher in the majority of mutants, whereas none showed substantially decreased variability (Figure 5B and Supplementary Figure 6A). Notably, plants do not always exhibit clear trends in increased difference from WT with cumulative mutations (Figures 5B,C and Supplementary Figure 6A).

For the plants with perturbed ABCB-mediated auxin transport, earlier organs of a similar phyllotactic index are more developed relative to the WT counterpart (Figure 5E), which correlates with the upregulated expression levels in organs of similar developmental stage (Supplementary Figure 5A). This, again, suggests a potential role of ABCB-mediated auxin efflux in regulating shoot morphology and early flower development.

## Discussion

We have presented a computational framework for analysing plant tissues on the organ-level in 3D. We have illustrated how our methods enable quantitative analysis of plant tissues using confocal data of high to intermediate resolution. The lack of dependence on single-cell segmentation quality data entails enriched possibilities for quantitative analysis even for data which lacks in quality. For example, it is possible to segment data lacking plasma membrane or cell wall dyes altogether, as well as plant tissues which have suffered damage that may affect tissue dyes, as long as some tissue-demarcating signal is present.

Whilst we have focused our exemplary analysis to 3D data of plant shoots, our framework is versatile, and has the potential to be applicable to tissues of varying morphologies (Supplementary Figure 3). We have here provided example implementations for the stages of preprocessing, contouring, surface generation, and segmentation, but our workflow is modular, and the different steps can be replaced and improved upon in future work. Nonetheless, our methodology used in this study illustrates the potential in investigating and phenotyping tissue-level data, especially in terms of geometric qualities. In the broader scope, our framework can be extended to allow for more versatile integration of fluorescent reporter data and to relate this to tissue-level substructures, such as individual flower organs.

In terms of the early flowers, the strong patterning in geometric data relative to the identified phyllotactic organ index in our analysis is indicative of these variables developing in a structured manner (Figure 4). As expected, the organ-level surface area follows a consistently increasing trend with increasing phyllotactic position, as does the distance of the corresponding organ centre of mass relative to the shoot apex. This indicates that within the scope of initiating primordia, a higher value in these variables generally indicates a more developed organ. Contrastingly, the increase in median vertex curvature relative to the first organs, followed by a negative trend for the subsequent organs is indicative of a more complex developmental trend. However, the overall coupling with the phyllotactic index implicates the median vertex curvature as a morphologically relevant variable in determining the organ developmental stage. The trend reversal itself appears to relate to the detachment of the organ from the meristem and initiation of the formation of the primordial pedicel (Figure 4; Smyth et al., 1990), and thus enables a highly reproducible, quantitative description of morphological events. However, it is important to note the importance of consistent data collection when connecting quantitative variables to morphological events, as including e.g., only partial flower tissues can obfuscate the analysis. Nevertheless, in the extended scope, our analysis could be used to link tissue-level development to the distribution patterns of relevant molecular regulators, which could help elucidate the regulatory mechanisms underpinning the formation of complex morphologies. The quantification of phyllotactic patterns in particular have often been done in late stages in order to generate sufficient statistics (Besnard et al., 2014). Whilst it remains difficult to capture more than a few initial flowers in a single confocal image, our high-throughput analysis pipeline puts the limitation on the data collection step, which, again, can be done faster when the analysis does not require the highest resolution when collecting the data.

The low error in fitting paraboloids to the WT segmented meristems indicate that the paraboloid is a good approximation of the inflorescence shape. In contrast, the high error in the mapping of a paraboloid to NPA-treated plants implicate that the overall structure is typically not well approximated by the apical meristem and the periphery and stem collectively in these plants, which may bear relevance to computational studies approximating the shoot with a quadric mathematical shape (Itoh et al., 2000; Bozorg et al., 2014; Gruel et al., 2016). In addition, this analysis may implicate a disconnect between the local growth determined by isotropic cell growth and proliferation in the central zone, and the apical-basal expansion of the cells in and below the rib meristem (Jacqmard et al., 2003). In contrast, the high correspondence between both the apex defined by the fitted paraboloid, as well as the inflorescence centre of mass, implicate a mutual dependence between the *CLV3* domain and the shoot geometry. This fits well with previous modelling predictions, where epidermal signals are part of regulating the positioning, and the curvature of the tissue itself becomes important (Gruel et al., 2016). Various methods have been presented in the literature to help identify the geometric apex of the meristem inflorescence (Willis et al., 2016; Galvan-Ampudia et al., 2020). Here, we have illustrated quantitatively how both the inflorescence centre of mass, as well as the paraboloid defined apex can approximate the *CLV3* defined apex with an error approximately equal to an average cell diameter in data of intermediate spatial resolution. In the extended scope, this provides researchers with the ability to parameterise the shoot surface. In conjunction with our segmentation method, this methodology can be used to represent spatial molecular patterning in terms of a shoot-specific coordinate system. In turn, this can aid researchers in determining how certain kinds of molecular patterning relates to and varies with the morphological stage of initiating flower primordia.

Our analysis of auxin transport mutants not canonically implicated as primary regulators of phyllotaxis unveils how phyllotactic robustness is partly a downstream consequence of plant-level growth and development, as perturbations in non-shoot localised auxin transporters still affect phyllotactic robustness (Figure 5C and Supplementary Figure 6A). The vast majority of mutant genotypes investigated exhibit higher standard deviations with respect to their intraplant divergence angles, as well as a higher frequency of singular aberrant divergence angles (Figure 5B). This finding is consistent with that Col-0, at large, is subject to constraining factors serving to optimise robustness in terms of organ positioning.

In terms of the auxin transport proteins present in the SAM, it is notable that perturbations of the peripherally expressed *ABCB19* (Supplementary Figure 5A) entails a smaller meristem relative to Col-0, as defined by the first-organ-distance, and that this effect is exacerbated with an additive mutation of *ABCB1* – an auxin efflux transporter known to be complementary of *ABCB19* (Bennett et al., 2016). In contrast, mutations of the *AUX1/LAX* genes, which are centrally and provascularly expressed (Bainbridge et al., 2008), exhibit an opposite, non-significant effect, with a larger shoot and potentially exacerbated effect with additive mutations (Figure 5D). A potential explanation for these respective phenotypes could relate to altered levels of auxin in the peripheral zone, and the highly sensitive phenotype identification presented here together with a more detailed combination of perturbations and a use of auxin reporters would provide a venue to investigate this further. Similarly, the apparent trend in the *ABCB1* and *19* genes both in terms of regulating shoot morphology (Figures 3A,C, 5D), and on early organ development (Figure 5E), suggests a potential role of long-range auxin transport proteins in regulating shoot development, especially considering its early organ-specific expression pattern (Supplementary Figure 5A). The methodology presented here provides an initial framework for exploring these phenotypes in further detail, both in terms of direct quantification of tissue-level geometries, and as a way of parameterising these shapes and connecting them further to abundance levels of relevant regulatory components.

The control of inflorescence dome shape at large remains obscure. However, we note that amongst the genes investigated in this study, perturbations of mechanically linked regulators tend to generally produce a stronger response in terms of shoot curvature asymmetry (Figure 3D). The result that mutations in the same gene families can give rise to both flatter and more pointed meristems suggests a dependency either on regionally specific expression domains or more complex feedback mechanisms in regulating the dome shape. The contrasting effect between mutations in the different *CesA* genes can be reconciled by taking their expression domains into account (Yang et al., 2016), which suggests that reduced mechanical stability in the inflorescence periphery leads to a flatter shape. However, it is not entirely clear how a uniformly reduced mechanical stability across the tissue would lead to a more pointed shoot. Similarly, whilst *XXT1* and *5* are uniformly expressed and *XXT2* is typically upregulated in flower primordia, both *xxt1,2* and *xxt1,2,5* exhibit a flatter dome (Figure 3B), potentially due to differentially weaker mechanical integrity in the peripheral initiation zones. This would suggest that not only the tissue robustness in absolute terms, but the relative mechanical integrity between the central and peripheral domain, is of importance. It is likely that future, theoretically grounded work can help address this issue in detail. Specifically, future studies may wish to further elucidate these phenotypes by assessing the impact of regional differences in mechanical properties, both in terms of absolute and relative cell wall rigidity, and on possible stress feedback on mechanical anisotropy particularly in the shoot periphery. The resulting consequences for cell growth and division dynamics, and the resulting influence on dome curvature could help elucidate how shoot morphology is regulated by cell-level properties and dynamics, similar to what has been done for single cells in e.g., pollen tube growth (Dumais et al., 2006), and bacteria studies (van Teeffelen et al., 2011).

In summary, our results herein have illustrated how computational methods can be used to quantify and segment tissue-level structures, particularly shoot apical meristems. Whilst our analysis is limited to a few, exemplary cases, our method is general and widely applicable, and can furthermore be extended and modified according to situational requirements. This allows researchers to access a new venue of data quantification and analysis, to help address fundamental questions in tissue development and morphodynamics in general.

## Materials and Methods

### Plant Material, Growth and Imaging Conditions

Plants belonging to the hormone transport dataset were incubated in cold (4°C) chambers for 3–5 days, sown on F2 Levington soil in PT40 trays, and kept in long-day conditions, i.e., 16/8 h light/dark cycles in 20°C, until between 24 and 28 days after germination. Plants in the mechanical mutant dataset were grown on soil as above under short day conditions, i.e., 8/16 h light/dark cycles in 20°C, for 4 weeks before being transferred to long day conditions as above until bolting.

Plants assuming bolting and reaching a stem height of 1–4 cm were decapitated and pruned to remove flower organs following published protocols (Prunet et al., 2016). Before imaging, plants were incubated in propidium iodide (Jones et al., 2016) or FM4-64 (Rigal et al., 2015) for a period of approximately 7–10 min, whereafter the shoots and imaging containers were washed thoroughly with water, and thereafter submerged in the same throughout the course of imaging.

Confocal data for plants grown on NPA conditions were acquired from previous studies (Willis et al., 2017). The soil condition comparison dataset consisted of plants containing *pCLV3::dsRed x myr-YFP* reporters, and were grown according to the hormone transport and mechanically perturbed datasets above, but were not stained due to the transgenic plasma membrane marker (Supplementary Table 1).

The origins of the *abcb1*, *abcb19*, *abcb1,19*, *pin3*, *pin4*, *pin7*, *pin3,4*, *pin3,7*, *pin4,7*, and *pin3,4,7* seeds have been described previously (van Rongen et al., 2019), as have *cesa1^any1^*, *cesa3^eli1^*, *cesa3^je5^*, *cesa6^prc1–1^*, *xxt1,2* and *xxt1,2,5*, (Fagard et al., 2000; Caño-Delgado et al., 2003; Desprez et al., 2007; Fujita et al., 2013; Xiao et al., 2016), *aux1* and *aux1lax1,2,3* (Bainbridge et al., 2008), and the *pCLV3::dsRed x myr-YFP* transgenic seeds (Willis et al., 2016; Supplementary Table 1).

Confocal stacks were acquired immediately after dissection at a resolution of approximately 0.5 × 0.55 × 0.55 μm^3^ per voxel using a 20x × /1.0 N.A. water immersion objective. Either a Zeiss LSM 880 or LSM 700 laser scanning confocal microscope was used for all collected data. Detailed imaging settings relating to each image are preserved in the accompanied image metadata.

In terms of the acquisition process, our protocol performs better in terms of accuracy for tissues acquired with high spatial resolution. However, this happens at the cost of computational efficiency. As such, it is often preferable to acquire images with sufficient resolution to easily discern the tissue outline by eye.

### Data Preprocessing

We used a number of preprocessing steps to improve image quality (Supplementary Figure 1). In order to improve signal quality, it is recommended to make use of deconvolution algorithms to compensate for optical distortion and to reduce noise (Wiener, 1949; Richardson, 1972; Lucy, 1974). However, in this study, we did not conduct this step due to sufficient data resolution. Further, as it is common for plant samples to suffer from variable signal quality across the tissue, often relating to signal or dye penetration artefacts, it is oftentimes appropriate to perform contrast adjustment in order to equalise the signal level. In our case, this was performed using Contrast Limited Adaptive Histogram Equalization (CLAHE; Pizer et al., 1987). In addition, conventional smoothing algorithms such as median and Gaussian filtering may be used to eliminate noise and even out the signal; here, we limited smoothing to Gaussian filtering. The choice of smoothing and noise elimination methods should be chosen relative to the data type and quality.

When working with large amounts of data with varying spatial properties, it can be helpful to transform the data to have the same spatial resolution, so as to minimise the need to tailor individual parameter sets to specific images. In our analysis herein, all original data was transformed using a third-order spline interpolation (Virtanen et al., 2020), to have an isometric voxel resolution of 0.5 μm in all spatial dimensions. This resolution was chosen manually to balance computational efficiency and output data quality. All original data and code including the preprocessing steps are available *via* open repositories (Data Availability).

In the mechanically perturbed dataset, not enough tissue was included to allow for reliable organ segmentation. Therefore, we instead manually cropped a cylindrical region around the CZ using ImageJ (Fiji^1^). The above processing steps were then applied.

### Contouring and Meshing

Contours of the input data were generated using the Morphological Chan-Vese algorithm (ACWE; Chan and Vese, 2001; Márquez-Neila et al., 2014), *via* the Scikit-Image framework (van der Walt et al., 2014). Further, several methods were implemented to fill in missed parts of the tissue interior following the ACWE step, which are provided in the code included with this study (Supplementary Figure 1). Specifically, we filled in holes in each XY section of the image, and extended the tissue contour downwards along the Z-dimension (to the lowest Z-coordinate) to fill in voxels beneath the tissue exterior. In certain cases of particularly bad data quality or signal artefacts, the ACWE algorithm proved unable of accurately distinguishing between interior and exterior parts of the tissue in question, where a simple thresholding mask was instead used to attain a representative contour; the threshold value used was calculated using Otsu’s method (Otsu, 1979) using the intensity data as input. The ACWE step is normally the most time-consuming part of the pipeline, taking approximately 5 min to process for a typical shoot apical meristem image on a Dell inspiron 15 7591 laptop (8GB DDR4, 2666 MHz RAM, Intel Core i7-9750H). For the NPA treated dataset, due to the available cellular segmentations, we utilised the non-background binary mask generated from the segmented images for generating the surfaces.

Fine-grained triangular meshes were generated using Lewiners’s Marching Cubes algorithm (Lewiner et al., 2003). The meshes were simplified using the Approximated Centroidal Voronoi Diagrams (ACVD) algorithm (Valette and Chassery, 2004), to get regular meshes with 10% of the original vertex density, although this parameter can be tuned according to preference. The downscaling parameter was chosen manually to balance output accuracy and computational efficiency. Further, in-house algorithms were utilised to ensure that all meshes were devoid of singularities, self-intersections and degenerate elements. Additionally, we heuristically remove aberrant mesh tissue through in-house algorithms. Mesh processing and management was performed predominantly using the Visualization ToolKit (VTK; Schroeder et al., 2000), largely through usage of the PyVista Python package (Sullivan and Kaszynski, 2019).

The mechanical dataset was generated with focus on the meristem proper and did not include sufficient flower tissue for accurate tissue segmentation. In these data, the segmentation step was therefore circumvented by cropping out an elliptical cylinder limited to the inflorescence central zone. The slightly differing surface generation method compared to the hormonal dataset illustrates the flexibility of the protocol.

### Curvature Computation

The mean curvature for all vertices in the meshes were generated using the PyVista package (Sullivan and Kaszynski, 2019). The scalar range of the curvature values were truncated to be within the range of (−0.1, 0.1) so as to avoid singularly obfuscating vertices; however, this truncation value can be adjusted as desired. Subsequently, iterative min-max filtering was applied on each vertex with its immediate neighbourhood, as defined by edge-connectivity, in order to amplify curvature peaks and lower curvature valleys. Lastly, iterative curvature averaging was applied in the same vein to smoothen the curvature field of the mesh.

### Segmentation

In order to segment the various substructures from the post-processed curvature field, an algorithm inspired by the Constanza protocol (Åhl et al., 2018; Bhatia et al., 2019) was implemented. For each vertex in the mesh, the neighbouring (i.e., connected by an edge) vertex with the highest curvature was thereafter identified. If no such vertices could be found, the current vertex was considered a curvature attractor. This process was repeated for all vertices in the mesh, and vertices with overlapping components were assigned a single integer label.

Following the initial segmentation, a series of post-processing steps were applied to merge labelling domains depending on certain properties. Implemented methods include (Supplementary Figure 1):

• Merge, depth: Merging of domains based on a scalar threshold criterion such that if the difference in curvature between the domain maximum and the maximum curvature value amongst the vertices bordering the neighbouring domain is below this value, the domains are merged. This ensures that all domains identified have at least the specified depth, which eliminates spurious, shallow domains.
• Merge, engulfing: Merging of domains based on whether more than a given threshold fraction of the domain boundary vertices borders another domain. This further removes spurious domains, and eliminates domains which are entirely contained within other domains.
• Merge, disconnected: Merging of a domain not connected to the inflorescence meristem (or specified domain) with its corresponding domain neighbour with the largest border, provided less than a the threshold fraction of the domain boundary vertices are connected to the inflorescence domain. This helps eliminate spurious domains, particularly in the image periphery, and assists with merging emerging sepals with their corresponding primordium.
• Merge, distance: Two domains are merged based on whether the Euclidean distance between their centre points is below a threshold value.
• Merge, angle: Merge two domains based on whether their respective centre points are within a threshold angle from each other relative to the inflorescence apex. This, again, helps merge emerging sepals with their corresponding flowers.

### Feature Identification and Data Extraction

The inflorescence domain is defined as the domain which is penetrated by a ray trace parallel to the z-axis and originates in the whole tissue centre of mass, or, alternatively, as the domain with the most neighbouring domains, which was done in cases when the centre of mass approach failed. Following the inflorescence identification, organs were manually ordered and a corresponding phyllotaxis spiral orientation determined, from which angles and positions could be extracted computationally. Specifically, angles were quantified relative to the domain centre points exclusively in the XY-plane. In turn, domain centre points were defined using either the centre of mass, where each vertex has a weight of 1, or alternatively in the case of the inflorescence meristem, by the apex point of a fitted paraboloid.

The paraboloid was defined by the equation

$$z=p_{1}\cdot x^{2}+p_{2}\cdot y^{2}+p_{3}\cdot x+p_{4}\cdot y+p_{5}$$

where *p*_*1*_ to *p*_*5*_ represent the defining parameters, and fitting the corresponding mesh vertices to this using the SciPy Least Squares optimiser (Virtanen et al., 2020). In order to improve the chances of a successful fit, up to five different initial parameter values were attempted. From the above, the paraboloid apex is defined by the coordinates

$$x=-p_{3}/\left(2\cdot p_{1}\right)$$

$$y=-p_{4}/\left(2\cdot p_{2}\right)$$

$$z=p_{1}\cdot x^{2}+p_{2}\cdot y^{2}+p_{3}\cdot x+p_{4}\cdot y+p_{5}.$$

The tissue-level Gaussian curvature is defined as the product of the principal curvature parameters, *p*_1_⋅*p*_2_, in the equations above, whereas the principal curvature ratio is defined as *max*({*p*_1_,*p*_2_})/*min*({*p*_1_,*p*_2_}). A mesh for the paraboloid is generated using the VTK implementation (Schroeder et al., 2000).

The *CLV3* apex is defined by computing the centre of mass of the voxels with higher CLV3 signal than the 99.99% image quantile. If no such voxels exist, the threshold is reduced first to the 99.90%, or lastly, the 99.00% quantile, and the computation is repeated.

Unless otherwise specified, all computations relating to the distance to the apex refer to the apex as specified by the inflorescence COM method. Accordingly, in the case of the first organ distance metric, we used the maximal Euclidean distance of all vertices in a given domain relative to the inflorescence apex.

### Statistical Tests Used and *p*-Value Annotation Table

*p*-values reported refer exclusively to Mann-Whitney-Wilcoxon (MWW) test, two-sided with Bonferroni correction. Statistical computations are done using the SciPy toolkit (Virtanen et al., 2020). We consistently use the following classifications of *p*-values:

ns: 5.00e-02 < *p* ≤ 1.00e+00
*: 1.00e-02 < *p* ≤ 5.00e-02
**: 1.00e-03 < *p* ≤ 1.00e-02
***: 1.00e-04 < *p* ≤ 1.00e-03
****: *p* ≤ 1.00e-04

## Data Availability Statement

The datasets and code used in this study can be found in online repositories. All original source data files used in this study are available *via* the Cambridge University Apollo Repository (https://doi.org/10.17863/CAM.82442). Confocal data for the leaf and anther images have been published previously (Wolny et al., 2020). All scripts and software for segmentation, quantification, analysis and visualisation are available *via* the Sainsbury Laboratory GitLab repository (https://gitlab.com/slcu/teamHJ/publications/aahl_etal_2022).

## Author Contributions

HÅ and HJ conceived and designed the presented research. HÅ and YZ collected data. HÅ wrote the initial draft of the manuscript and developed the code used in this study and performed computations and analysis. HÅ and HJ wrote the final version of the manuscript with input from YZ. HJ provided funding and supervision. All authors analysed and discussed the results and contributed to the final manuscript.

## Conflict of Interest

The authors declare that the research was conducted in the absence of any commercial or financial relationships that could be construed as a potential conflict of interest.

## Publisher’s Note

All claims expressed in this article are solely those of the authors and do not necessarily represent those of their affiliated organizations, or those of the publisher, the editors and the reviewers. Any product that may be evaluated in this article, or claim that may be made by its manufacturer, is not guaranteed or endorsed by the publisher.

## Footnotes

1
https://fiji.sc

## Supplementary Material

Supplementary Movie 1 Inflorescence shape-approximation through paraboloid-fitting. Example illustration of a paraboloid (green) fitted to the inflorescence domain (brown), extracted from the full shoot (blue). The paraboloid is fitted using a least squares fit on the vertices in the inflorescence surface, minimising the error relative to the corresponding input for a paraboloid (Methods).
