# Supplementary material for: High-Throughput 3D Phenotyping of Plant Shoot Apical Meristems From Tissue-Resolution Data
Source: Front Plant Sci. 2022 Apr 18;13:827147. doi: 10.3389/fpls.2022.827147 (PMC9062647; doi:10.3389/fpls.2022.827147)
Supplement: Supplementary file 1 [file Data_Sheet_1.pdf]

*Supplementary Material*

# 1 Supplementary Figures and Tables

## 1.1 Supplementary Figures

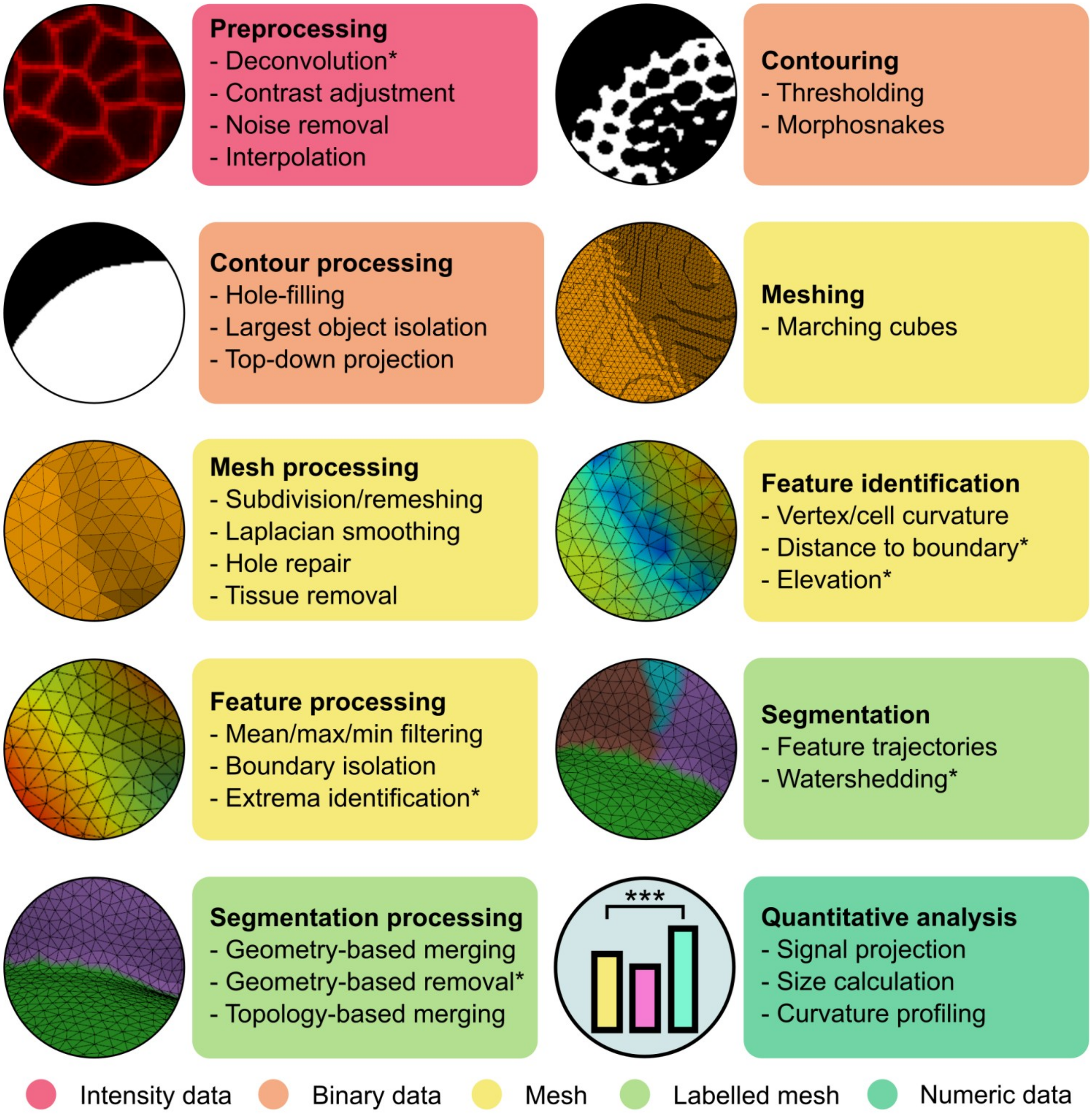


**Supplementary Figure 1: Graphical illustration of the quantification pipeline including example methods pertaining to the corresponding processing step.** The categories are performed in order left to right order, top to bottom. Each category textbox lists exemplary steps which can be performed in relation to each processing category. Sub-steps marked with an asterisk (*) were not performed in this study. Circular inset graphics show examples of the data type and appearance subsequent to the corresponding processing step. For a detailed description of the processing steps, see Methods.

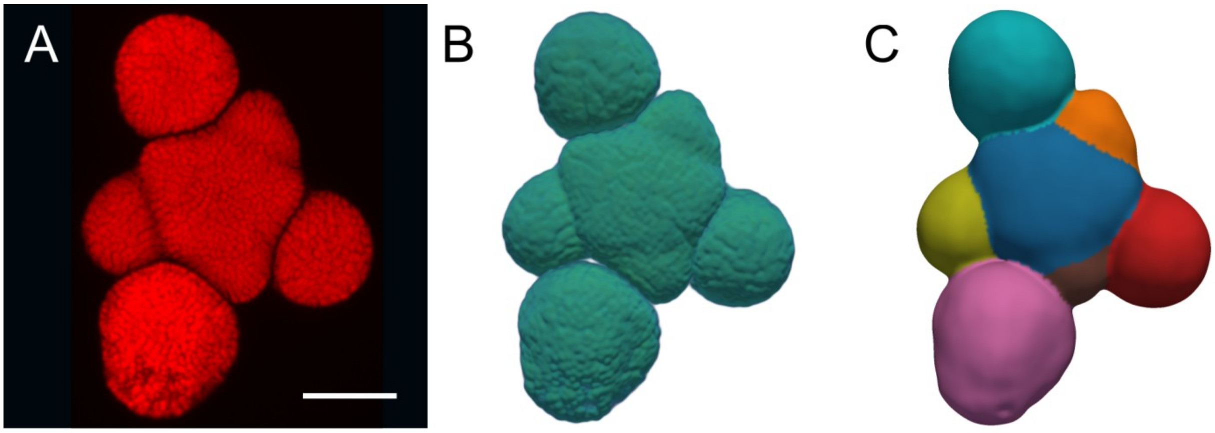


**Supplementary Figure 2: Successful tissue-level segmentation of injured plant tissue**
Illustrative example of a successful segmentation of an injured *Arabidopsis thaliana* SAM. **(A)** Summed projection of the raw confocal data. The shoot was injured during acquisition, and the subsequent propidium iodide staining dyed the entire cells, rendering an opaque tissue. Consequently, single-cell segmentation would not have been possible. Scale bar: 75 µm. **(B)** 3D Volume render of the raw confocal data. **(C)** Successful surface segmentation of the tissue substructures following application of our protocol (Methods). Individual substructures are coloured according to their corresponding segmentation label. Scale in **(B-C)** further to **(A)**. Colouring in **(A-B)** shows the corresponding signal intensity magnitude in arbitrary units; **(C)** is coloured by integer label value.

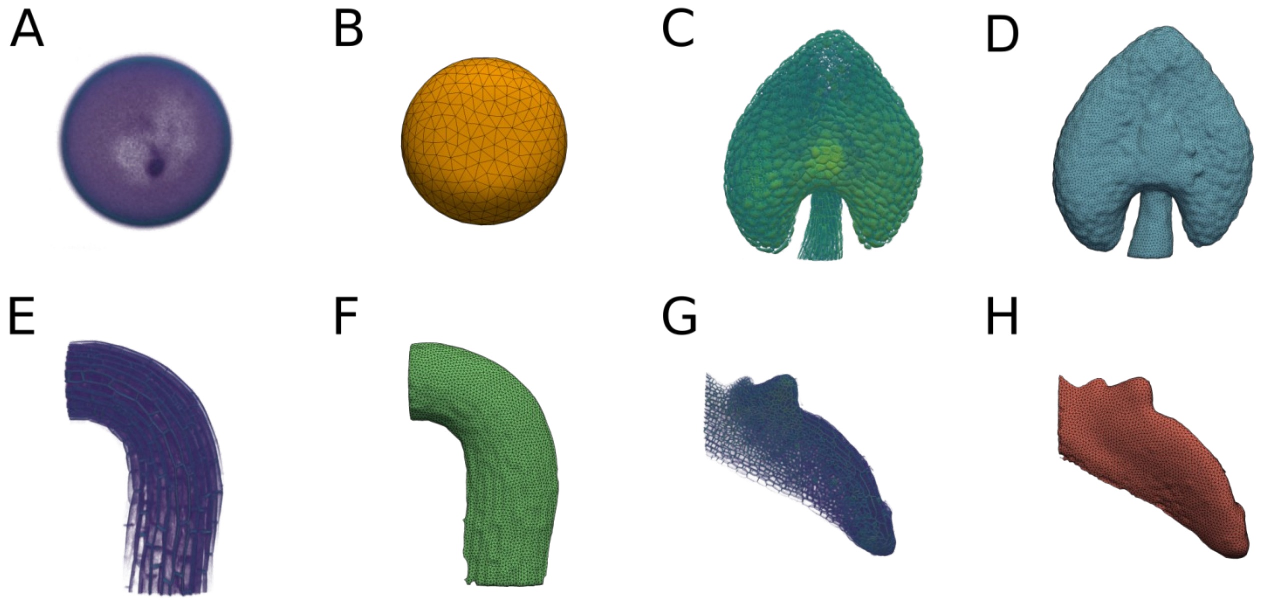


**Supplementary Figure 3: Illustration of capabilities for surface generation on a diverse set of plant tissues**
Collection of raw confocal data and generated surfaces for a collection of different plant tissues. **(A-B)** Protoplast and corresponding surface; **(C-D)** Anther and corresponding surface. **(E-F)** Apical hook and corresponding surface. **(G-H)** Leaf blade and corresponding surface. Approximate scales in terms of maximal extent, left to right: **(A-B)** 10 µm, **(C-D)** 350 µm, **(E-F)** 300 µm, **(G-H)** 400 µm. Colouring in **(A, C, E, H)** shows the signal intensity magnitude in arbitrary units; **(B, D, F, H)** are false-coloured. For data origins, see Data Availability Statement.

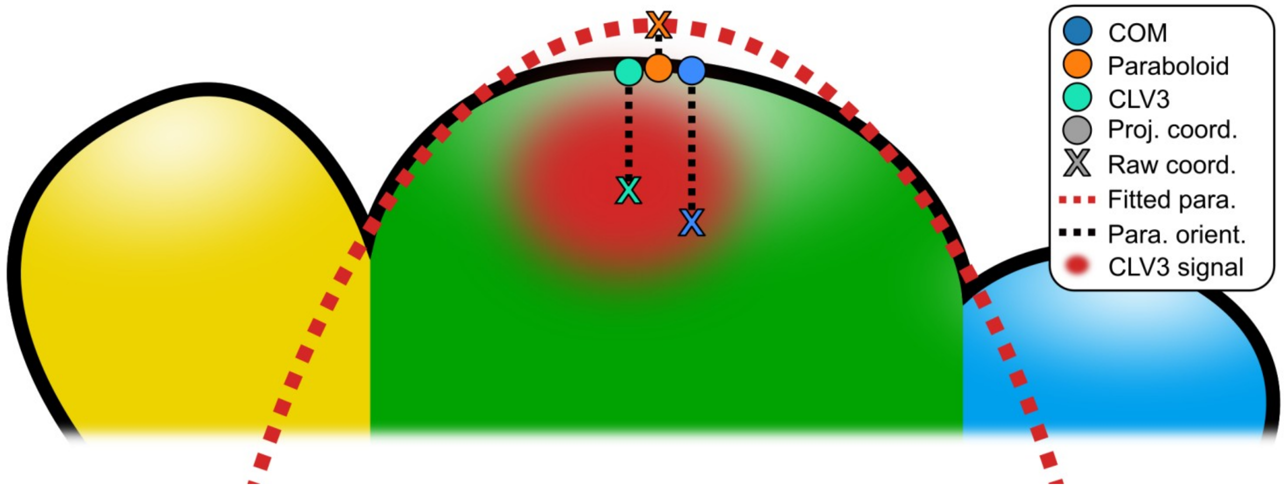


**Supplementary Figure 4: Conceptual 2D illustration of the apex coordinate quantification framework**
Graphical illustration of the plant shoot. A paraboloid is fitted to a surface mesh of the inflorescence meristem, and the corresponding apex coordinates are subsequently derived by projection to the mesh (Methods). In wild-type plants, the tissue is first segmented to remove flower organs (yellow and blue), and isolate the SAM (green); in NPA-treated plants, no initial organ-removal is needed (Methods). The paraboloid fit is exaggerated for illustrative purposes.

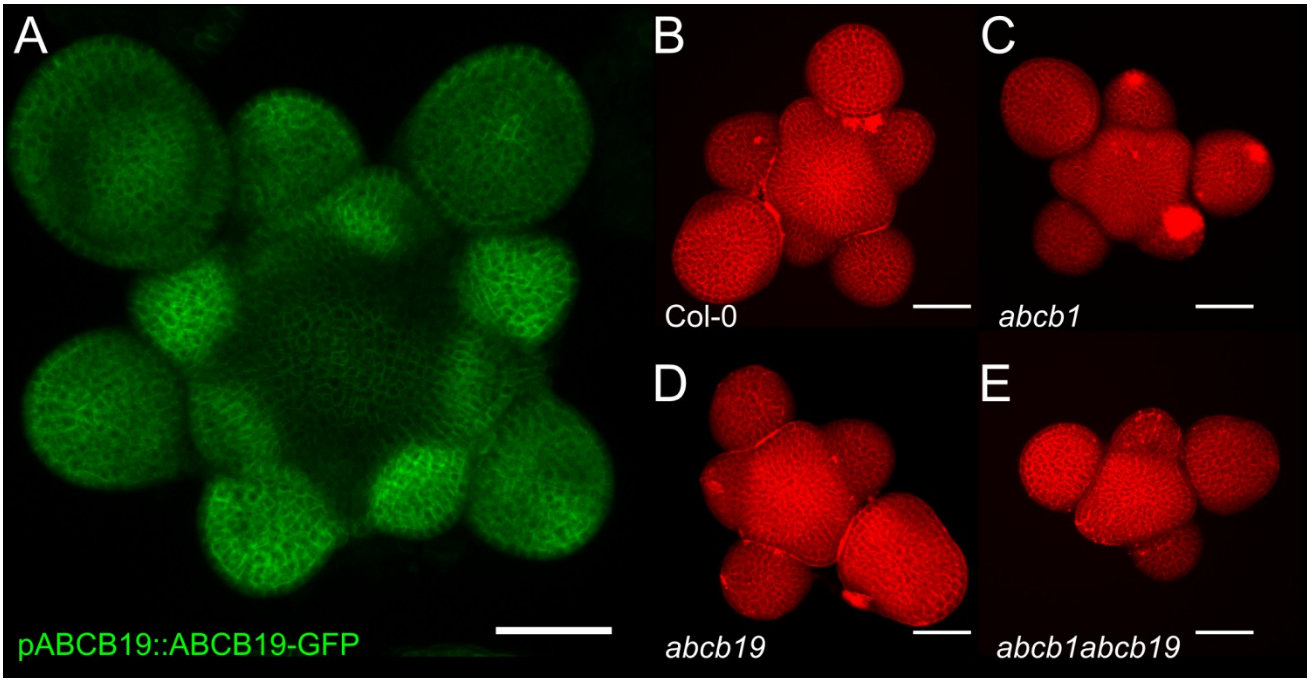


**Supplementary Figure 5: *ABCB19* expression is upregulated in early flower primordia**
**(A)** Summed projection in the apical-basal direction of fluorescent expression of a SAM expressing *pABCB19::ABCB19-GFP*. Early flower primordia of developmental stage 1 to early stage 3 exhibit upregulated expression. Wassilewskija ecotype. **(B-E)** Same as **(A)**, but for propidium iodide stained plants, illustrating the shoot morphology in representative Col-0 **(B)**, *abcb1* **(C)**, *abcb19* **(D)** and *abcb1abcb19* **(E)** plants. Mutations in the *ABCB* gene family perturb the shoot morphology relative to the wild type plants. Scale bars: 50 μm.

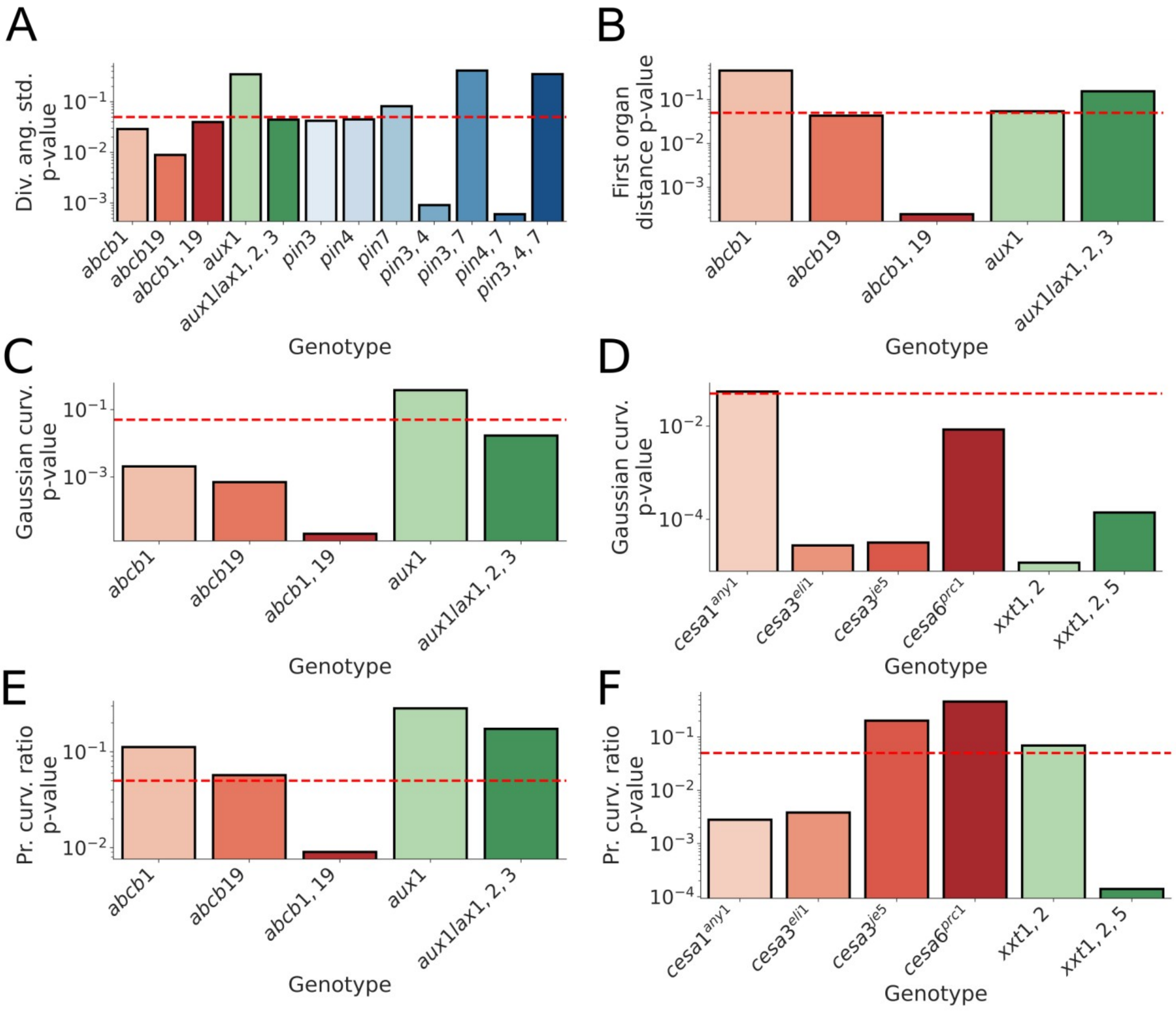


**Supplementary Figure 6: P-value outcome for significance tests between genotype distributions**
Corresponding p-values for the distributions of various statistical variables, namely the **(A)** Divergence angle standard deviation for the extended auxin transport dataset; **(B)** First organ distance for the auxin transport dataset; **(C)** Gaussian curvatures for the auxin transport dataset; **(D)** Gaussian curvatures for the mechanical dataset; **(E)** Principal curvatures for the auxin transport dataset; **(F)** Principal curvatures for the mechanical dataset. All statistical tests refer to MWW tests (Methods). Red dashed lines are refer to the canonical significance threshold, p = 0.05 (*).

## 1.2 Supplementary Tables

**Supplementary Table 1: Plant material, abbreviations and origin**

| Genotype | In-text abbreviation | Reference |
|---|---|---|
| *Col-0* | | |
| *abcb1* | | van Rongen et al., 2019 |
| *abcb19* | | van Rongen et al., 2019 |
| *abcb1abcb19* | *abcb1,19* | van Rongen et al., 2019 |
| *aux1* | | Bainbridge et al., 2008 |
| *aux1lax1lax2lax3* | *aux1lax1,2,3* | Bainbridge et al., 2008 |
| *pin3* | | van Rongen et al., 2019 |
| *pin4* | | van Rongen et al., 2019 |
| *pin7* | | van Rongen et al., 2019 |
| *pin3pin4* | *pin3,4* | van Rongen et al., 2019 |
| *pin3pin7* | *pin3,7* | van Rongen et al., 2019 |
| *pin4pin7* | *pin4,7* | van Rongen et al., 2019 |
| *pin3pin4pin7* | *pin3,4,7* | van Rongen et al., 2019 |
| $cesa1^{any1}$ | | Fujita et al., 2013 |
| $cesa3^{eli1}$ | | Caño-Delgado et al., 2003 |
| $cesa3^{je5}$ | | Desprez et al., 2007 |
| $cesa6^{prc1\text{-}1}$ | | Fagard et al., 2000 |
| *xxt1xxt2* | *xxt1,2* | Xiao et al., 2016 |
| *xxt1xxt2xxt5* | *xxt1,2,5* | Xiao et al., 2016 |
| *pCLV3::dsRed x pUBQ10::myr-YFP* | *pCLV3::dsRed x myr-YFP* | Willis et al., 2016 |
| *pABCB19::ABCB19-GFP* | | van Rongen et al., 2019 |
